# Surgical Management for Early-Stage Bilateral Breast Cancer Patients in China

**DOI:** 10.1371/journal.pone.0122692

**Published:** 2015-04-13

**Authors:** Jia-jian Chen, Nai-si Huang, Jing-yan Xue, Chen-lian Quan, Yu-long Tan, Guang-yu Liu, Zhi-min Shao, Jiong Wu

**Affiliations:** 1 Department of Breast Surgery, Fudan University Shanghai Cancer Center, Shanghai, China; 2 Department of Oncology, Shanghai Medical College, Fudan University, Shanghai, China; The First Affiliated Hospital with Nanjing Medical University, CHINA

## Abstract

**Background:**

The aim of this study was to investigate the current surgical management strategy for bilateral breast cancer (BBC) patients and to assess the changes in this strategy in China.

**Methods:**

This is a retrospective review of all patients with early-stage BBC who underwent surgical treatment at the Fudan University Shanghai Cancer Center between June 2007 and June 2014.

**Results:**

A total of 15,337 patients with primary breast cancer were identified. Of these patients, 218 (1.5%) suffered from synchronous bilateral breast cancer (sBBC), and 296 (2.0%) suffered from metachronous bilateral breast cancer (mBBC). Patients with a lobular carcinoma component, those with estrogen receptor-positive cancer, and those with an accompanying sclerosing adenosis in the affected breast tended to develop BBC. The rates of bilateral mastectomy, breast conserving therapy, reconstruction, and combined surgeries were 86.2%, 6.4%, 3.7%, and 3.7%, respectively, for patients with sBBC and 81.1%, 4.4%, 3.0%, and 11.5%, respectively, for patients with mBBC. The interval between bilateral cancers, age at first diagnosis of breast cancer, histopathological type, and stage have significant impacts on the choice of surgery for patients with BBC.

**Conclusions:**

Bilateral mastectomy was the dominant surgical management for patients with BBC in China, despite the increased application of breast reconstruction surgery observed in recent years. Bilateral prosthetic breast reconstruction was the ideal choice for patients with sBBC. Chinese surgeons should take responsibility for patient education and inform their patients about their surgical options.

## Introduction

The optimal type of surgery for breast cancer patients continues to be a controversial topic. Revolutionary changes in the surgical management of breast cancer occurred during the 20^th^ century, from radical to minimal surgery [1], and these changes were supported by several landmark trials with decades of follow-up. Breast-conserving therapy (BCT) accompanied by radiation therapy is associated with a survival rate that is equivalent to that of mastectomy for selected breast cancer patients [2, 3]. For patients who are not candidates for BCT or who choose not to conserve, skin-sparing mastectomy with breast reconstruction is a safe technique that provides a better cosmetic outcome without compromising oncological safety [4, 5]. In addition, sentinel lymph node biopsy (SLNB) has been adopted as an alternative to axillary lymph node dissection (ALND) for node staging [6, 7], with improved postoperative quality of life for node-negative patients [8].

In the United States, a recent population-based retrospective study revealed a 13.5% decrease in the application of mastectomy alone and a 42.0% increase in the application of immediate breast reconstruction [9]. In contrast, based on a nationwide survey in China, mastectomy still remains the dominant option for the surgical treatment of breast cancer [10]. However, for patients with bilateral breast cancer (BBC), including both synchronous bilateral breast cancer (sBBC) and metachronous bilateral breast cancer (mBBC), the surgical management strategies are relatively more complicated. When determining the surgical management for bilateral breast cancer patients, each breast must be considered individually to determine the optimal surgical management, and the symmetry and postoperative appearance of the breasts should also be taken into consideration.

Due to the improved diagnostic technologies and management strategies, an increasing number of women are at risk for developing BBC [11]. Different types of surgeries might be associated with different levels of psychological distress for patients with BBC [12]. Therefore, more attention should be devoted to the surgical management of patients with BBC. The present study aimed to investigate the current surgical management strategy for patients with BBC and to assess changes in this strategy in a retrospective series of patients in China.

## Materials and Methods

### Patients

Patients diagnosed and treated with operable BBC at Fudan University Shanghai Cancer Center between June 2007 and June 2014 were enrolled in this retrospective analysis. A time interval of 12 months between bilateral breast cancers was introduced to distinguish sBBC (≤12 months) and mBBC (>12 months) according to our previous study [11]. The clinicopathological and epidemiological parameters of each patient were obtained from electronic medical records.

Patients treated with operable unilateral breast cancer (UBC) during the same time period served as the control group. Male patients and stage IV patients who underwent palliative operations were excluded from the study. In addition, to avoid the risk of misclassifying metastatic bilateral breast disease, patients with stage IIIb or IIIc (T4 or N3) disease were also excluded from the present study.

The protocol of the present retrospective study was approved by the Ethics Committee of Fudan University Shanghai Cancer Center. A written informed consent form allowing the academic application of de-identified photographs and medical records was obtained from each patient.

### Statistical Analysis

The independent samples t test and ANOVA were performed to compare continuous variables, while Fisher’s exact test and the Pearson chi-square test were used to analyze categorical variables. The Kappa consistency test was applied to evaluate the relationship between the interval between bilateral cancers and the types of surgeries. All results with p<0.05 were considered statistically significant (SPSS statistical analysis program, version 20.0; SPSS Inc., Chicago, IL, USA).

## Results

Between June 2007 and June 2014, 15,337 patients with primary breast cancer underwent surgeries at the Fudan University Shanghai Cancer Center. Of these patients, 218 (1.5%) suffered from sBBC, and 296 (2.0%) suffered from mBBC. The patient demographics and clinicopathological characteristics are described in Table 1.

**Table 1:** Patient demographics and clinicopathological characteristics.

Variables	sBBC	mBBC	UBC	p
Age (median, range)	53 (28–89)	1st 47 (20–75)	51 (18–98)	<0.001
		2nd 57 (22–89)		<0.001
Family history	20	43	1333	0.023
Histopathology *				<0.001
Ductal	188	259	12759	
Lobular	14	14	217	
Others	16	23	856	
Stage †				0.076
0	24	31	1695	
I	57	100	3756	
II	116	133	7232	
IIIa	21	32	1149	
Type of surgery				<0.001
Mastectomy	188	240	12018	
Breast Conserving Therapy	14	13	2705	
Breast Reconstruction	8	9	614	
Combined	8	34	0	
ER-positive ‡	159/192	188/235	6384/8629	0.003
Her-2-positive ‡	30/164	51/213	1960/9410	0.542
Accompanying sclerosing adenosis	23	11	121	<0.001

sBBC, synchronous bilateral breast cancer; mBBC, metachronous bilateral breast cancer; UBC, unilateral breast cancer.

*Carcinomas with a lobular carcinoma component in either breast were classified as *lobular*, while those with the involvement of other carcinoma components, with the exception of ductal or lobular carcinoma, in either breast were classified as *others*.

† Due to a lack of data, the first carcinoma could not be staged in 45.9% of the patients with mBBC.

‡ Invasive cancer was classified based on ER or Her-2 expression in either breast.

### Differences in the clinicopathological characteristics of patients with BBC and patients with UBC

Patients with mBBC developed the first carcinoma at an earlier age compared to patients with UBC (47 vs. 51, p<0.001), while patients with sBBC were significantly older than those with UBC (53 vs. 51, p<0.001). Patients with mBBC were more likely to have a family history of first-degree relatives with breast cancer compared to patients with UBC (p = 0.004). Consistent with our previous results [11], patients with a lobular carcinoma component, those with estrogen receptor (ER)-positive cancer, and those with an accompanying sclerosing adenosis in the affected breast tended to develop sBBC (Table 1). In addition, the present study confirmed that these patients also tended to develop mBBC. No significant differences were found in the stage distribution, the percentage of ER-positive patients, or the percentage of Her-2-positive patients among the patients with sBBC, mBBC and UBC (Table 1).

### Distribution of surgeries among BBC patients

**Fig 1:**
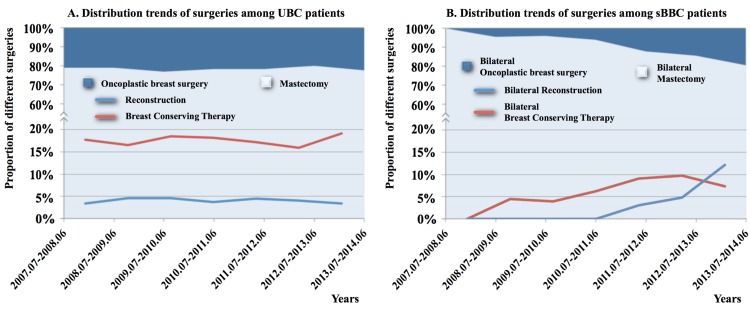
Distribution trends of the different types of surgeries. (A) Distribution trends of surgeries among patients with unilateral breast cancer. (B) Distribution trends of surgeries among patients with synchronous bilateral breast cancer. The light blue area indicates the percentage of patents who underwent unilateral (A) or bilateral (B) mastectomy. The dark blue area indicates the percentage of patients who received unilateral (A) or bilateral (B) oncoplastic surgeries, including breast conserving therapy (red line) and breast reconstruction techniques (blue line), in each year.

**Fig 2:**
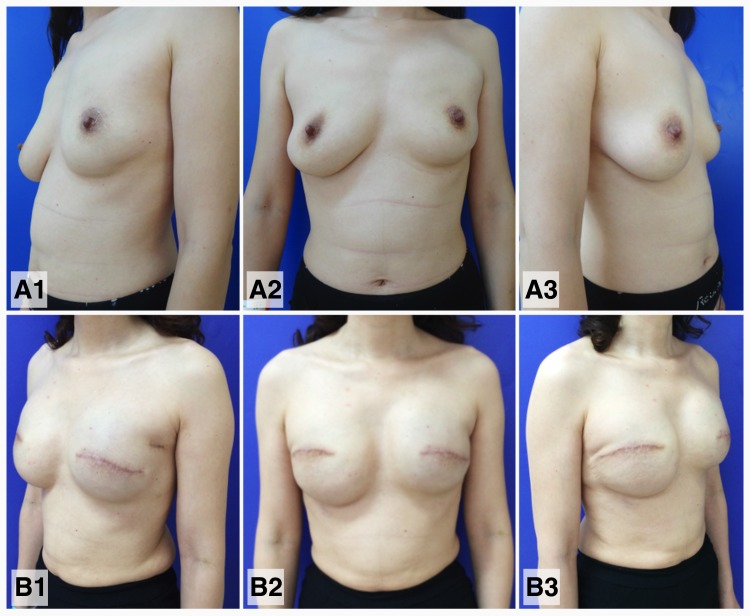
Bilateral prosthetic breast reconstruction for a patient with synchronous bilateral breast cancer. A 42-year-old patient was diagnosed with synchronous bilateral lobular carcinoma in situ on April 22, 2012. Bilateral mastectomy and tissue expander-implant breast reconstruction were performed. (A1-3) A preoperative view of the breasts; (B1-3) a 10-month postoperative view of the breasts after breast reconstruction.

The rates of bilateral mastectomy, BCT, reconstruction, and combined surgeries (BCT or reconstruction in one breast and mastectomy in the contralateral breast) were 86.2%, 6.4%, 3.7%, and 3.7%, respectively, for patients with sBBC and 81.1%, 4.4%, 3.0%, and 11.5%, respectively for patients with mBBC. The rates of mastectomy, BCT, and reconstruction for patients with UBC were 78.4%, 17.6%, and 4.0%, respectively (Table 1). The distributions of the different types of surgeries performed for patients with UBC remained stable over the years; however, the distributions changed significantly over the years for patients with sBBC (Fig 1). In fact, an apparent paradigm shift in the different types of surgeries performed for patients with sBBC was observed. The application of BCT has increased gradually in recent years, while breast reconstruction therapy has rapidly increased in popularity in recent years. Of the eight patients with sBBC who underwent skin-sparing mastectomy and immediate breast reconstruction, seven patients (87.5%) underwent reconstruction with the tissue expander-implant technique (Fig 2), and one patient underwent BCT for one breast and reconstruction with transverse rectus abdominis myocutaneous flap for the contralateral breast.

Of the 296 patients with mBBC, 261 (88.2%) underwent mastectomy of the initial breast cancer. Of these, 240 (92.0%) patients also underwent mastectomy and 16 patients (6.1%) underwent BCT when they developed the second primary breast cancer. The remaining five (1.9%) patients underwent skin-sparing mastectomy on the second primary breast cancer followed by bilateral breast reconstruction. In addition, another four patients underwent skin-sparing mastectomy followed by immediate breast reconstruction for the first and second primary tumors. Breast reconstruction was used at a similar rate to treat the first primary tumor in patients with mBBC and patients with UBC (4.1% vs. 4.0%, p = 0.881). Of the 28 patients with mBBC who underwent BCT for the first tumor, 15 (53.6%) patients underwent mastectomy for the second primary tumor, and the remaining 13 (46.4%) patients underwent BCT.

### Choice of surgery among BBC patients

Among the 514 patients with BBC, the interval between bilateral cancers, age at first diagnosis of breast cancer, histopathological type, and stage had a significant impact on the choice of surgery among patients with BBC (Table 2). Patients with sBBC or those with mBBC with an interval between bilateral cancers of over 10 years had a significant tendency to undergo bilateral mastectomy, with mastectomy rates of 86.2% and 93.9%, respectively. Patients with lobular cancer in either of the breasts were also more likely to undergo bilateral mastectomies (with a rate of 89.3%) compared to those without a lobular carcinoma component (p = 0.035). The ER status and Her-2 status had no impact on the choice of surgery among patients with BBC (Table 2).

**Table 2:** Association between clinicopathological characteristics and different types of surgeries.

	Mastectomy	Oncoplastic breast surgery	Combined surgery	p
Interval between bilateral cancers				<0.001
0–1 year	188	22	8	
1–5 years	72	11	18	
5–10 years	75	9	12	
>10 years	93	2	4	
Age (median, range)	51 (26–79)	43 (20–80)	47 (30–89)	<0.001
Histopathology				0.035
Ductal	377	35	35	
Lobular	25	1	2	
Others	26	8	5	
Stage				0.001
0	51	4	1	
I	123	21	13	
II	213	18	17	
IIIa	41	1	11	
ER-positive	352/422	39/43	32/40	0.918
Her-2-positive	113/426	12/40	6/40	0.397

## Discussion

A recently published study reviewed the status of breast cancer in China with respect to its epidemiology, diagnosis and treatment [13]. However, the current status of BBC management was not mentioned in this study. Thus, the present study fills this knowledge gap by exploring the surgical management of patients with BBC in China.

### Bilateral mastectomy: the most common choice of surgical management for BBC patients in China

Although the rate of mastectomy among patients with UBC in the present study was significantly lower than that previously published in a hospital-based, nationwide, multi-center retrospective study in China (78.4% vs. 92.3%, p<0.001) [10], it was still significantly higher than the rates in Europe and the Americas [9, 14]. The average breast volume of Chinese women is significantly smaller than that of European and American women [15, 16], which resulted a smaller proportion of breast cancer patients that were indicated for BCT. In addition, the deep-rooted traditional beliefs regarding cancer, such as the firm belief among the Chinese population that all types of cancer should be maximally resected, along with the lack of the patient education may also prompt a subset of breast cancer patients who would be suitable for BCT to refuse the appropriate surgery.

The likelihood that patients with sBBC would undergo bilateral conserving surgery was even lower than the likelihood that patients with UBC would undergo this surgery; however, both patients and surgeons should take unilateral or bilateral breast reconstruction into consideration when determining the treatment strategy. A recent study evaluated trends and variations in the use of breast reconstruction among patients with breast cancer undergoing mastectomy in the United States. This study indicated that the proportion of patients undergoing bilateral mastectomy significantly increased from 3% in 1998 to 18% in 2007 (p<0.001), and these patients were more likely to undergo reconstruction (p<0.001) [14]. However, in China, the mastectomy rate among patients with sBBC was significantly higher than that among patients with UBC (approximately 86.2%; p = 0.003), while the reconstruction rate among patients with sBBC was only 3.7% (Table 1); this rate is even lower than the rate among patients with UBC (4.0%; p = 0.491). A major reason for this finding is that the demand for an optimal postoperative cosmetic appearance is lower among Chinese patients, reflecting the influence of deep-rooted traditional concepts and a lack of knowledge about breast cancer therapeutics. In the present study, eight (3.7%) patients received mastectomy on one breast and BCT on the contralateral breast, which further suggested that cosmetic appearance and breast symmetry were not important to these patients. The surgeons should also take responsibility for the rather high mastectomy rate because they are responsible for educating and informing their patients about surgical options. A previously published survey of Chinese breast cancer patients’ opinions regarding BCT indicated that the patients’ level of understanding of BCT and the suggestions of doctors and spouses have a significant impact on the decision-making process regarding the type of surgery (p<0.05) [17]. Additional effort should be devoted to improving public awareness and knowledge about breast cancer in China.

The mastectomy rate was higher among patients with mBBC than among patients with UBC (p = 0.146) (Table 1). The median time of surgery for the first primary tumor in patients with mBBC was 7.6 years ahead of that in patients with UBC, which may account for the finding that significantly fewer patients with mBBC underwent BCT for the first developed breast cancer compared to patients with UBC (9.5% vs. 17.6%, p = 0.001). The mastectomy rate was significantly more closely related with the interval between bilateral cancers (κ = 0.104, p<0.001) (Table 2). The mastectomy rate was 93.9% for patients with an interval of over 10 years between bilateral cancers, and this rate was 71.3% when the interval was less than five years. A possible explanation for this finding was that the patients’ satisfaction regarding their current quality of life increased as the time interval from the cancer diagnosis increased, while the demand for a better cosmetic appearance when selecting the type of surgery for the secondary cancer was reduced as the time interval from the cancer diagnosis increased.

### The use of bilateral breast reconstruction for the surgical management of BBC patients in China is becoming more common

In theory, compared to patients with UBC, patients with sBBC may be more likely to want to preserve the cosmetic appearance of the breasts to avoid the severe psychological trauma caused by the removal of both breasts during the operation [18]. Patients might also be more satisfied with bilateral reconstruction because of improved symmetry, superior aesthetic appearance without clothing, and overall satisfaction with the reconstructive process [19]. However, the rate of breast reconstruction was lower among patients with sBBC than among patients with UBC in China, although breast reconstruction has become more popular among patients with sBBC in recent years (Fig 1).

Recent studies focused on the paradigm shift in breast reconstruction in the United States have suggested that changes in mastectomy patterns (i.e., 15% and 12% yearly increases in the application of contralateral and bilateral prophylactic mastectomies) have resulted in a dramatic increase in the application of prosthetic breast reconstruction (i.e., from 40% to 74%) [20, 21]. In the present study, the elevated application of bilateral breast reconstruction in patients with sBBC was also due to the promotion of bilateral prosthetic breast reconstruction in recent years. Prosthetic breast reconstruction could be safely performed in patients who received skin-sparing mastectomy and has the advantages of a shorter operative time, shorter hospitalization, shorter recovery time, less complex surgery, and fewer complications at the donor site compared with autologous breast reconstruction, which was also considered by surgeons when selecting the reconstruction method [14]. Almost all patients who received bilateral skin-sparing mastectomy were candidates for bilateral prosthetic breast reconstruction, and the use of this method could provide a satisfactory cosmetic appearance (Fig 2). In addition, the costs of skin-sparing mastectomy and immediate bilateral breast reconstruction with prosthesis (not including the cost of implants or tissue expanders), pedicle flaps, and free flaps were approximately $810, $1,100 and $1,295, respectively. These similar costs might also partially explain the preference for prosthetic reconstruction compared with autologous reconstruction.

**Fig 3:**
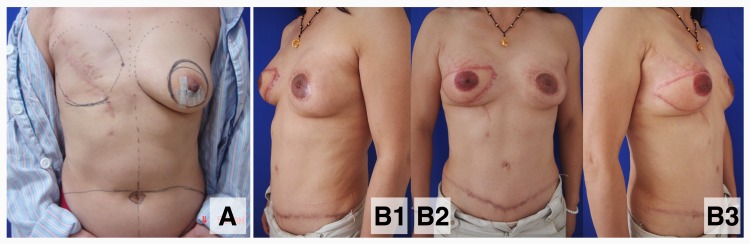
Bilateral autologous breast reconstruction for a patient with metachronous bilateral breast cancer. A 46-year-old patient diagnosed with metachronous bilateral breast cancer. Mastectomy was previously performed on April 15, 2006 for invasive ductal carcinoma in the right breast. A second primary invasive ductal carcinoma was diagnosed on December 24, 2010, and a skin-sparing mastectomy with immediate reconstruction with a deep inferior epigastric perforator flap was performed on the left breast. This procedure was accompanied by a delayed reconstruction with a superficial inferior epigastric artery flap performed on the right breast. Bilateral nipple-areola reconstruction and tattooing was performed 9 months later. (A) A frontal view of the patient before management of the second primary tumor; (B1-3) an 18-month postoperative view after breast reconstruction.

For patients with mBBC, an even lower rate of breast reconstruction was observed. The development of a second primary tumor in the contralateral breast could have a tremendous psychological impact on the patients, which could present in various forms. In the present study, among the patients who had received bilateral breast reconstruction, over half underwent mastectomy when the first tumor occurred and chose bilateral breast reconstruction when the second tumor developed. Delayed autologous breast reconstruction alone with immediate prosthetic reconstruction or bilateral autologous breast reconstruction were the major procedures used for reconstruction for mBBC patients (Fig 3).

In addition, the present study further confirmed our previous result that patients with a lobular carcinoma component, those with ER-positive cancer, and those with an accompanying sclerosing adenosis in the affected breast tended to develop sBBC [11]. The limitations of the present study included the potentially limited external validity of results from a single institution. The results of the present study might overestimate the current status of surgical management of patients with early-stage BBC in China. In addition, community-related parameters such as marriage, fertility, health insurance, level of education, average annual earnings, and geographical distribution of the patients were absent from the analysis. Further sociological and cost-effect analyses might account for the non-medical factors associated with the current status of surgical management for patients with BBC in China.

## Conclusion

The present study provided an overview of the current state of surgical management for early-stage patients with BBC in China. Bilateral mastectomy was the dominant surgical management for patients with BBC in China, despite the increased use of breast reconstruction observed in recent years. Bilateral prosthetic breast reconstruction was the ideal choice for patients with sBBC. More efforts are required to improve public awareness and knowledge about the disease in China, while surgeons should also take responsibility for patient education and for informing their patients about surgical options.

## References

[1] Cotlar AM, Dubose JJ, Rose DM. History of surgery for breast cancer: radical to the sublime. Current surgery. 2003;60(3):329–37. 14972270 10.1016/S0149-7944(02)00777-8

[2] Black DM, Hunt KK, Mittendorf EA. Long term outcomes reporting the safety of breast conserving therapy compared to mastectomy: 20-year results of EORTC 10801. Gland surgery. 2013;2(3):120–3. 10.3978/j.issn.2227-684X.2013.06.01 25083471 PMC4115749

[3] Fisher B, Anderson S, Bryant J, Margolese RG, Deutsch M, Fisher ER, et al Twenty-year follow-up of a randomized trial comparing total mastectomy, lumpectomy, and lumpectomy plus irradiation for the treatment of invasive breast cancer. The New England journal of medicine. 2002;347(16):1233–41. 12393820 10.1056/NEJMoa022152

[4] Agrawal A, Sibbering DM, Courtney CA. Skin sparing mastectomy and immediate breast reconstruction: a review. European journal of surgical oncology: the journal of the European Society of Surgical Oncology and the British Association of Surgical Oncology. 2013;39(4):320–8. 10.1016/j.ejso.2012.12.015 23333068

[5] Tan BK, Chim H, Ng ZY, Ong KW. Aesthetic design of skin-sparing mastectomy incisions for immediate autologous tissue breast reconstruction in asian women. Archives of plastic surgery. 2014;41(4):366–73. 10.5999/aps.2014.41.4.366 25075359 PMC4113696

[6] Chen JJ, Wu J. Management strategy of early-stage breast cancer patients with a positive sentinel lymph node: With or without axillary lymph node dissection. Critical reviews in oncology/hematology. 2011;79(3):293–301. 10.1016/j.critrevonc.2010.06.008 20663684

[7] Lyman GH, Temin S, Edge SB, Newman LA, Turner RR, Weaver DL, et al Sentinel lymph node biopsy for patients with early-stage breast cancer: American Society of Clinical Oncology clinical practice guideline update. Journal of clinical oncology: official journal of the American Society of Clinical Oncology. 2014;32(13):1365–83. 24663048 10.1200/JCO.2013.54.1177

[8] Chen JJ, Huang XY, Liu ZB, Chen TW, Cheng JY, Yang WT, et al Sentinel node biopsy and quality of life measures in a Chinese population. European journal of surgical oncology: the journal of the European Society of Surgical Oncology and the British Association of Surgical Oncology. 2009;35(9):921–7. 10.1016/j.ejso.2009.01.009 19233602

[9] Zhong T, Fernandes KA, Saskin R, Sutradhar R, Platt J, Beber BA, et al Barriers to immediate breast reconstruction in the Canadian universal health care system. Journal of clinical oncology: official journal of the American Society of Clinical Oncology. 2014;32(20):2133–41. 24888814 10.1200/JCO.2013.53.0774

[10] Li J, Zhang BN, Fan JH, Pang Y, Zhang P, Wang SL, et al A nation-wide multicenter 10-year (1999–2008) retrospective clinical epidemiological study of female breast cancer in China. BMC cancer. 2011;11:364 10.1186/1471-2407-11-364 21859480 PMC3178543

[11] Chen JJ, Wang Y, Xue JY, Chen Y, Chen YL, Xiao Q, et al A clinicopathological study of early-stage synchronous bilateral breast cancer: a retrospective evaluation and prospective validation of potential risk factors. PloS one. 2014;9(4):e95185 10.1371/journal.pone.0095185 24736632 PMC3988153

[12] Schubart JR, Emerich M, Farnan M, Stanley Smith J, Kauffman GL, Kass RB. Screening for Psychological Distress in Surgical Breast Cancer Patients. Annals of surgical oncology. 2014. 10.1245/s10434-014-3919-8 25034820

[13] Fan L, Strasser-Weippl K, Li JJ, St Louis J, Finkelstein DM, Yu KD, et al Breast cancer in China. The lancet oncology. 2014;15(7):e279–89. 10.1016/S1470-2045(13)70567-9 24872111

[14] Jagsi R, Jiang J, Momoh AO, Alderman A, Giordano SH, Buchholz TA, et al Trends and variation in use of breast reconstruction in patients with breast cancer undergoing mastectomy in the United States. Journal of clinical oncology: official journal of the American Society of Clinical Oncology. 2014;32(9):919–26. 24550418 10.1200/JCO.2013.52.2284 PMC4876312

[15] Qiao Q, Zhou G, Ling Y. Breast volume measurement in young Chinese women and clinical applications. Aesthetic plastic surgery. 1997;21(5):362–8. 9299007 10.1007/s002669900139

[16] Kayar R, Civelek S, Cobanoglu M, Gungor O, Catal H, Emiroglu M. Five methods of breast volume measurement: a comparative study of measurements of specimen volume in 30 mastectomy cases. Breast cancer: basic and clinical research. 2011;5:43–52. 10.4137/BCBCR.S6128 21494401 PMC3076010

[17] Zhang L, Jiang M, Zhou Y, Du XB, Yao WX, Yan X, et al Survey on breast cancer patients in China toward breast-conserving surgery. Psycho-oncology. 2012;21(5):488–95. 10.1002/pon.1922 21322089

[18] Roostaeian J, Crisera C. Current options in breast reconstruction with or without radiotherapy. Current opinion in obstetrics & gynecology. 2011;23(1):44–50. 20978439 10.1097/GCO.0b013e328340e18a

[19] Sinno S, Salvino MJ, Vandevender D. Comparing patient satisfaction in bilateral and unilateral breast reconstruction. Plastic surgical nursing: official journal of the American Society of Plastic and Reconstructive Surgical Nurses. 2014;34(3):141–5; quiz 6–7. 25188854 10.1097/PSN.0000000000000048

[20] Albornoz CR, Bach PB, Mehrara BJ, Disa JJ, Pusic AL, McCarthy CM, et al A paradigm shift in U.S. Breast reconstruction: increasing implant rates. Plastic and reconstructive surgery. 2013;131(1):15–23. 10.1097/PRS.0b013e3182729cde 23271515

[21] Cemal Y, Albornoz CR, Disa JJ, McCarthy CM, Mehrara BJ, Pusic AL, et al A paradigm shift in U.S. breast reconstruction: Part 2. The influence of changing mastectomy patterns on reconstructive rate and method. Plastic and reconstructive surgery. 2013;131(3):320e–6e. 10.1097/PRS.0b013e31827cf576 23446580

